# Functional Annotation of All Salmonid Genomes (FAASG): an international initiative supporting future salmonid research, conservation and aquaculture

**DOI:** 10.1186/s12864-017-3862-8

**Published:** 2017-06-27

**Authors:** Daniel J. Macqueen, Craig R. Primmer, Ross D. Houston, Barbara F. Nowak, Louis Bernatchez, Steinar Bergseth, William S. Davidson, Cristian Gallardo-Escárate, Tom Goldammer, Yann Guiguen, Patricia Iturra, James W. Kijas, Ben F. Koop, Sigbjørn Lien, Alejandro Maass, Samuel A. M. Martin, Philip McGinnity, Martin Montecino, Kerry A. Naish, Krista M. Nichols, Kristinn Ólafsson, Stig W. Omholt, Yniv Palti, Graham S. Plastow, Caird E. Rexroad, Matthew L. Rise, Rachael J. Ritchie, Simen R. Sandve, Patricia M. Schulte, Alfredo Tello, Rodrigo Vidal, Jon Olav Vik, Anna Wargelius, José Manuel Yáñez, The FAASG Consortium, Craig R. Primmer, Daniel J. Macqueen, Ross D. Houston, Barbara F. Nowak, Willie S. Davidson, Sigbjørn Lien, Ben F. Koop

**Affiliations:** 1 Institute of Biological and Environmental Sciences, University of Aberdeen, Aberdeen, AB24 2TZ UK; 2 Department of Biology, University of Turku, 20014 Turku, Finland; 3 The Roslin Institute and Royal (Dick) School of Veterinary Studies, The University of Edinburgh, Midlothian, EH25 9RG UK; 4 Institute for Marine and Antarctic Studies, University of Tasmania, Launceston, TAS Australia; 5 Département de biologie, Institut de Biologie Intégrative et des Systèmes (IBIS), Université Laval, Québec, G1V 0A6 Canada; 6 The Research Council of Norway, Drammensveien 288, P.O. Box 564, NO-1327 Lysaker, Norway; 7 Department of Molecular Biology and Biochemistry, Simon Fraser University, Burnaby, BC V5A 1S6 Canada; 8 Laboratory of Biotechnology and Aquatic Genomics, Interdisciplinary Center for Aquaculture Research, Department of Oceanography, Universidad de Concepción, 4030000 Concepción, Chile; 9 Leibniz Institute for Farm Animal Biology, Institute for Genome Biology, Fish Genetics Unit, Wilhelm-Stahl-Allee 2, 18196, Dummerstorf, Germany; 10 INRA, UR1037 Fish Physiology and Genomics, Rennes, France; 11 Human Genetics Program ICBM Faculty of Medicine, University of Chile, Santiago, Chile; 12 CSIRO Agriculture, QLD, St Lucia, 4067 Australia; 13 Department of Biology, University of Victoria, Victoria, BC V8W 3N5 Canada; 14 Centre for Integrative Genetics (CIGENE), Department of Animal and Aquacultural Sciences, Norwegian University of Life Sciences, NO-1432 Ås, Norway; 15 Center for Mathematical Modelling, Department of Mathematical Engineering, University of Chile, 8370456 Santiago, Chile; 16 Center for Genome Regulation, University of Chile, 8370456 Santiago, Chile; 17 School of Biological, Earth and Environmental Sciences, University College Cork, Cork, Ireland; 18 Center for Biomedical Research, Universidad Andres Bello, 8370146 Santiago, Chile; 19 FONDAP Center for Genome Regulation, Faculty of Biological Sciences and Faculty of Medicine, Universidad Andres Bello, 8370146 Santiago, Chile; 20 School of Aquatic and Fishery Sciences, University of Washington, Box 355020, Seattle, WA 98195 USA; 21 Conservation Biology Division, Northwest Fisheries Science Center, National Marine Fisheries Service, National Oceanic and Atmospheric Administration, 2725 Montlake Blvd E, Seattle, WA 98112 USA; 22 Matis Ltd, Vínlandsleið 12, 113 Reykjavík, Iceland; 23 NTNU - Norwegian University of Science and Technology, NO-7491 Trondheim, Norway; 24 National Center for Cool and Cold Water Aquaculture, USDA ARS, 11861 Leetown Road, Kearneysville, WV 25430 USA; 25 Department of Agricultural, Food, and Nutritional Science, University of Alberta, Edmonton, AB Canada; 26 Office of National Programs, USDA ARS, 5601 Sunnyside Avenue, Beltsville, MD 20705-5148 USA; 27 Department of Ocean Sciences, Memorial University of Newfoundland, 1 Marine Lab Road, St. John’s, NL A1C 5S7 Canada; 28 Genome British Columbia, Suite 400 – 575, West 8th Avenue, Vancouver, BC V5Z 0C4 Canada; 29 Department of Zoology, University of British Columbia, 6270 University Blvd, Vancouver, BC V6T 1Z4 Canada; 30 Instituto Tecnológico del Salmón S.A., INTESAL de SalmonChile, Puerto Montt, Chile; 31 Laboratory of Molecular Ecology, Genomics, and Evolutionary Studies, Department of Biology, University of Santiago, 9170022 Santiago, Chile; 32 Institute of Marine Research, P.O. Box 1870, Nordnes, NO-5817 Bergen, Norway; 33 Faculty of Veterinary and Animal Sciences, University of Chile, Av. Santa Rosa 11735, Santiago, Chile & Aquainnovo, Cardonal s/n, Puerto Montt, Chile

**Keywords:** Salmonid fish, Genome biology, Functional annotation, Comparative biology, Standardized data and metadata, Data sharing, Aquaculture, Whole genome duplication, Evolution, Phenotyping

## Abstract

We describe an emerging initiative - the ‘Functional Annotation of All Salmonid Genomes’ (FAASG), which will leverage the extensive trait diversity that has evolved since a whole genome duplication event in the salmonid ancestor, to develop an integrative understanding of the functional genomic basis of phenotypic variation. The outcomes of FAASG will have diverse applications, ranging from improved understanding of genome evolution, to improving the efficiency and sustainability of aquaculture production, supporting the future of fundamental and applied research in an iconic fish lineage of major societal importance.

**Electronic supplementary material:**

The online version of this article (doi:10.1186/s12864-017-3862-8) contains supplementary material, which is available to authorized users.

## The importance of salmonid fishes: from evolution to sustainable food production

Salmonids have combined scientific, societal and economic importance that is unique among fish (reviewed in [1]). They are naturally distributed in fresh and marine habitats throughout the Northern hemisphere and have been introduced to South America, Australia, Africa and the Middle East. They perform key ecological functions, e.g. [2], but many populations are declining, and extensive effort is being directed towards their conservation and management, especially with respect to anthropogenic-driven change, e.g. [3]. Salmonids include at least 70 species (but are sometimes classified as >200), possessing a rich diversity of adaptations and life-history strategies [4]. The great phenotypic diversity amongst salmonids provides an excellent study system to understand adaptive divergence and ecological speciation [4, 5] and was potentially facilitated by a whole genome duplication (WGD) in their common ancestor ~95 Mya [6, 7]. Salmonid aquaculture and capture fisheries (mainly of Atlantic salmon *Salmo salar* L. and *Oncorhynchus* spp.) play an important role in the economic and/or food security of several nations, accounting for 7.2/16.6% of all traded fish in terms of share by weight/value [8].

## Rationale for the FAASG initiative

**Fig. 1:**
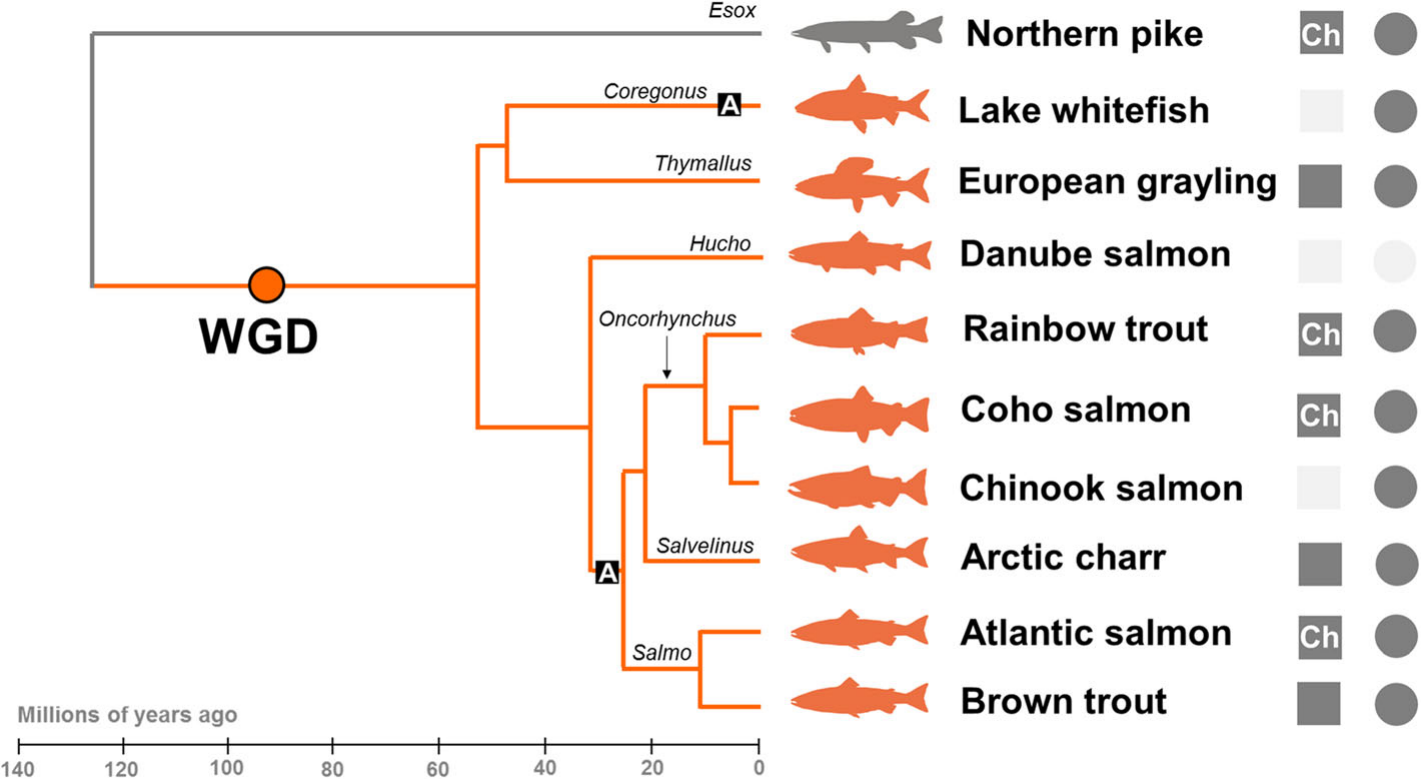
The comparative-evolutionary framework of FAASG. Shown are the initial target species for functional annotation (see Table 1) and their evolutionary relationships (time-calibrated tree after [7]). The selected species come from all three salmonid subfamilies. The position of the salmonid-specific WGD is highlighted (after [7, 9, 10]), along with Latin names of genera. Additional salmonid species that are future potential targets for functional annotation are not shown. Two lineages where anadromous life-history is thought to have evolved independently are highlighted ‘A’ (after [47]). The status of genomics resources are shown to the right of the tree: squares and circles indicate genome and transcriptome assemblies, respectively (*dark grey* = resource either published or close to being published; *light grey* = resource under active development; ‘Ch’ = chromosome-anchored genome assembly)

The FAASG initiative follows the recent publication of the genomes of Atlantic salmon [9] and rainbow trout (*Oncorhynchus mykiss*) [10], which have proved invaluable to salmonid researchers (section Genome-led science in salmonids: progress, challenges and unresolved questions) and establish a solid foundation for generating reference genome sequences for other salmonid species (Fig. 1). The next step for salmonid research is to annotate genome function, considering species and populations of major scientific interest (sections The FAASG framework, Data and assays). This will lay foundations to understand how genotypes are translated to phenotypes via different layers of regulation of gene and protein expression. Covering a broad diversity of research in salmonid biology will aid this action and is best achieved by involving the widest possible research community (section Operational structure, funding and research community engagement). FAASG will follow principles established by the ‘Functional Annotation of Animal Genomes’ (FAANG) consortium (section Rationale for linking with FAANG) [11], a similar international consortium initiative aimed at producing comprehensive maps of functional elements in terrestrial livestock genomes. This will include use of standardized approaches for functional annotation, including bioinformatics protocols and pipelines exploiting knowledge from other species and through an array of experimental assays (Table 1, section Data and assays). However, the FAASG framework (section The FAASG framework, Fig. 1) will also exploit unique features of salmonid biology, including recent WGD and extensive phenotypic variation at both macro- and micro-evolutionary timescales, to generate broad mechanistic insights into genome evolution and adaptation.

**Table 1:** Levels of genome-wide functional annotation within the FAASG framework

Class of variation	Context	Origin of data	Goal
Genomic sequence	Phylogeny-wide	Comparative analysis	Define fixed substitutions across species including for WGD gene duplicates. Assign to different classes: exonic, intronic, regulatory, synonymous vs. non-synonymous; radical vs. conservative non-synonymous and divergent from ancestral state Identify differences in structural genomic variation among species and describe its evolution Associate sequence/structural genome variation with epigenetic, transcriptomic and proteomic variation
Genomic sequence	Population-level	Genome-resequencing	Define SNPs and structural genome variation within species. Assign to different classes: as above Associate sequence/structural genome variation with epigenetic, transcriptomic and proteomic variation
Epigenetic (DNA methylation)	Phylogeny-wide and population level	Assays described in Additional file 1: Table S1.	Generate DNA methylome maps and define their regulation across tissues, developmental stages and common-garden physiological manipulations Associate changes in methylation with all forms of genomic, transcriptomic, proteomic and other classes of epigenetic variation
Epigenetic (histone modifications)	Phylogeny-wide and population level	Assays described in Additional file 1: Table S1	Define a range of histone marks and their regulation across tissues, developmental stages and common-garden physiological manipulations Associate variation in histone marks with all forms of genomic, transcriptomic, proteomic and other classes of epigenetic variation
Epigenetic (chromatin biology)	Phylogeny-wide and population level	Assays described in Additional file 1: Table S1	Generate maps of DNA accessibility and define their regulation across tissues, developmental stages and common-garden physiological manipulations Associate changes in chromatin structure with all forms of genomic, transcriptomic, proteomic and other classes of epigenetic variation
RNA expression	Phylogeny-wide and population level	RNAseq - potentially stranded protocols (see Additional file 1: Table S1)	Define expression of miRNA, mRNA and non-coding RNA across adult tissues, developmental stages and common-garden physiological manipulations [1] Associate transcriptomic variation to all forms of genomic, epigenetic and proteomic variation
Protein level	Phylogeny-wide and population level	Various possible mass spectrometer platforms – bottom up approach	Define proteome across tissues, developmental stages and common-garden physiological manipulations Associate proteomic variation to all forms of genomic, transcriptomic and epigenetic variation

## Genome-led science in salmonids: progress, challenges and unresolved questions

Notable progress in understanding of salmonid biology has stemmed from sequencing two salmonid genomes, as well as that of northern pike *Esox lucius* [12], a sister lineage that did not undergo the salmonid-specific WGD (Fig. 1). Genome-wide analyses have offered key insights into the remodelling and divergence of duplicated genome content and functions during the post-WGD rediploidization process [9, 10]. Population genomics has been revolutionized by genotyping-by-sequencing, whole genome re-sequencing and high-density SNP arrays [13–15], used for example to discover SNPs near the *vgll3* gene that explain 40% of the variation in sea-age at maturity [16, 17], genomic variation explaining the timing of migration [18] and adaptive population differentiation in immune function [19]. Population genomics is now routinely applied in salmonids without a genome sequence, by exploiting conserved synteny with rainbow trout or Atlantic salmon, e.g. [20–23]. Genome-wide approaches have also been applied to improve the accuracy of selection for key production traits (e.g. disease resistance) in breeding programs, either through genomic selection [24–26] or by characterization of major effect loci, e.g. [27, 28]. Further, the salmonid and pike genomes have been used to progress understanding of salmonid phylogeny and species diversification [7] and facilitate characterization of the molecular basis and post-WGD evolution of several physiological systems, including smoltification [29], growth [30], immunity [19, 31, 32] and olfaction [33]. Finally, the recent demonstration of successful genome editing in salmonids for gene knockout [34–37] opens the door for validation of candidate functional genomic elements and causative polymorphisms. Genome editing also has potential to address certain challenges in aquaculture, by creating new alleles and introducing them to farmed populations, and by expediting the selection of existing beneficial alleles [38].

Nonetheless, salmonid research and its applications have only just begun to exploit the possibilities of genome-led science. Undoubtedly, a number of unresolved questions and important challenges can be addressed through the FAASG initiative (Table 2).

**Table 2:** The role of functional genome annotation in addressing key challenges for salmonid research and its application. Below we list selected key questions, highlight their importance, and then briefly describe (*in italics*) how the FAASG initiative will help address them

Aquaculture
What is the functional genetic basis of key performance traits for salmonid aquaculture?
Few causative variants underlying performance trait QTL have been identified. Knowledge of the precise functional variants underpinning QTL will inform the biology of these traits, and facilitate cost-effective selection for favorable alleles. *Genome annotation is essential to prioritize candidate causal variants. Many traits are influenced by non-coding variants influencing gene expression. The FAASG initiative will aid identification and prioritization of QTL-region variants for key traits.*
How can we optimize genomic selection for genetic improvement in aquaculture breeding programs?
Genomic selection can accelerate genetic gain for traits important to sustainable and profitable aquaculture, such as host resistance to infectious diseases. Predicting breeding values in distant relatives to the training population is challenging, thus necessitating frequent, expensive phenotypic tests. *The likelihood of SNPs having a functional effect on a trait can be estimated using FAASG functional annotation data. These SNPs can be prioritized in genotyping panels to enable improved prediction accuracy, and persistency of that accuracy, across diverse genetic backgrounds and multiple generations.*
What is the functional genetic basis of recent domestication in salmonid species?
Salmonids are excellent models to study the genomic basis of recent domestication, facilitating discovery of genetic variation of importance in adaptation to aquaculture environments. These outcomes can improve hatchery management, health and welfare of farmed fish, and have implications for interactions with wild populations. *Domestication is likely to have a polygenic basis and be largely due to modification of gene regulation including control by epigenetic mechanisms. Functional annotation is essential for researchers to identify sequence and epigenomic variation linked to domestication and the response to artificial selection.*
How can genome editing technology contribute to improved aquaculture production?
Genome editing technology, notably CRISPR-Cas9 has potential to enhance aquaculture production directly by introducing favorable alleles into farmed populations, or indirectly, for example by providing a better understanding of the functional basis of production traits (e.g. using gene knockout). While regulatory and public acceptance is required, the potential is highlighted by several high profile successes in terrestrial livestock. *Choosing the correct target to edit is essential, and requires accurate annotation of the reference genome. A function of a SNP, epigenetic mark, non-coding RNA, coding RNA or whole protein can be determined using gene editing. The technology can also be applied to demonstrate causality of variants underlying QTL.*
What is the long term impact of aquaculture escapees on wild populations?
Evaluating and understanding the impacts of aquaculture escapees on wild populations supports risk assessment for the use of native and non-native strains in culture. *FAASG will improve understanding of the functional differences among populations resulting from genomic variation, and will guide development of tools to effectively track and monitor the genetic impact of escapees on wild populations.*
How can measurement of salmonid health and welfare in aquaculture be improved?
Appropriate biomarkers of stress, health and growth status in salmonid aquaculture are currently difficult to define and far from comprehensive. *An improved understanding of the genetic and epigenetic regulation of key physiological systems supporting fish health will be guided by the annotated genomes, networks and comparative biology, and will facilitate development of tools to help monitor animal wellbeing in culture.*
Ecology, evolution and physiology
What role did the whole genome duplication and subsequent rediploidization play in salmonid evolution?
This is a long-standing question of fundamental importance to our understanding of salmonid biology and the role of WGDs in evolution more generally. *Comparative genomic annotation will improve understanding of how sequence and functional variation arising post-WGD are coupled to trait evolution, including the lineage-specific evolution of anadromous life-history, which has been linked to species radiation.*
How important is genetic vs. epigenetic variation in regulating trait variability?
Rapid phenotypic divergence and phenotypic plasticity are hallmarks of many salmonid species, yet remain poorly-characterized. An improved understanding of heritable epigenetic variation and its interaction with both genetic and environmental variation can be exploited in both conservation and aquaculture. *Functional annotation of epigenetic marks in salmonid genomes, and studies into the role of epigenetic regulation in determining trait variation and phenotypic plasticity are key goals of the FAASG initiative.*
What is the genomic basis of response and adaptation to natural and anthropogenic stressors?
Human-induced environmental changes, including climate change, are already negatively affecting salmonid populations. Understanding the role of genetic and epigenetic variation in physiological response to these changes will be key to predicting, and potentially mitigating, these effects. *Improved understanding of the functional genomic basis of differential responses to environmental stressors in salmonids may be applied to inform forecasting, mitigation and remedial strategies for challenges associated with anthropogenic-induced changes in ecosystems, including through climate change.*
What role do ‘non-coding’ RNAs have in generating phenotypic variation?
The functions of non-coding RNAs are poorly understood in salmonids. The greater retention of miRNAs in comparison to duplicated genes after WGD suggests important functions in coping with a duplicated genome. Non-coding RNAs may regulate traits of interest to aquaculture and evolutionary biology. *Comparative functional annotation in salmonids will highlight the location and role of non-coding RNAs in regulation of gene expression and downstream regulation of complex traits.*
How many salmonid species exist, and how can we distinguish them?
The actual number of salmonid species is unknown. Habitat-dependent phenotypes can suggest different species, but genomics and functional genomics methods are ultimately required to answer this question. *Diverse salmonid species and populations will be targeted in FAASG, providing comparative genome sequences and annotations. This will facilitate development and application of species-specific markers to assess the quantity and diversity of species in the salmonid family.*

## Traits of crosscutting relevance: from aquaculture to evolution (and beyond)

Several traits of importance to aquaculture show extensive natural variation among salmonid species and populations, including disease resistance, growth rate, the control of sexual determination and maturation, and the physiological transition from fresh to saltwater. These traits have crosscutting relevance to multiple scientific fields, both fundamental and applied, and the dissection of their functional genomic architecture under the FAASG initiative will help address challenges faced by the aquaculture sector, along with long-standing research questions. Accordingly, the outcomes of FAASG will facilitate selection of aquaculture strains with improved disease resistance and higher product quality that reach market earlier [39–41], while explaining the evolutionary role of trait variation in wild populations [16, 42, 43] and informing management actions influencing population resilience, conservation, and re-introduction [23, 44–46]. Comparing the outcomes of artificial vs. natural selection on functional pathways under different conditions will also help dissect the genetic architecture of traits. For example, different populations will often share genetic variation influencing a trait, but aquaculture and wild conditions impose divergent selective pressures, leading to unique, yet complementary opportunities to understand natural selection and domestication.

## Rationale for linking with FAANG

The FAANG consortium aims to produce comprehensive maps of the functional elements in the genomes of domesticated animal species [11], building on the ENCODE project [47]. Underpinning principles of both consortia include use of robust, standardized experimental protocols based on defined tissues or cell types. These principles apply to both ‘wet lab’ experiments and bioinformatic analyses of data, which provides a comprehensive and reliable resource available for use by a wide research community. The FAASG initiative will link to FAANG, adhere to these principles, and utilise and build on the FAANG protocols and pipelines to avoid redundancy. FAANG is focussed on livestock species with high-quality reference genomes (chicken, pig, cattle and sheep), but with scope for inclusion of other species. The initial focus of FAASG will be the key farmed salmonids (Atlantic salmon and rainbow trout), but will expand to a broader range of lineages of interest to conservation, management and evolution (Fig. 1). In doing so, the initiative will harness wider diversity within a comparative context (section The FAASG framework) to understand the evolution of functional genome elements following species radiation and WGD. FAASG will provide a FAANG-type model for other species and lineages with recently-developed genome assemblies, the number of which is rapidly increasing. This includes other species of importance for global aquaculture and food security, for example tilapia, carp, catfish and shellfish species. There will also be great scope for cross-talk between FAASG and research communities for model fish species where functional annotation is advanced, including zebrafish *Danio rerio* (https://zfin.org/). All data generated via FAASG-linked projects will be made publicly available in a timely manner, in keeping with the principles of FAANG. More specifically, the consortium is committed to the release of all data produced in an open access manner, rapidly and before publication, in adherence with the standards defined in the FAASG Data Sharing Statement (https://www.faasg.org/data-sharing-principle/), which includes both the Toronto Statement about pre-publication data sharing, and the Fort Lauderdale principles about the release of data and materials prior to publication.

## The FAASG framework

The initial approach of FAASG will exploit a rich phylogenetic framework, documenting functionally important sequence variation and data derived from a core set of experimental assays (section Data and assays) across nine salmonid species and the northern pike (Fig. 1), under experimental conditions representative of the traits listed in section Traits of crosscutting relevance: from aquaculture to evolution (and beyond). Salmonid species were selected on the basis that genome sequencing projects are underway within the research community and represent six out of nine true genera from all three subfamilies, namely Salmoninae (*Salmo*, *Oncorhynchus*, *Salvelinus* and *Hucho*), Thymallinae (*Thymallus*) and Coregoninae (*Coregonus*) (Fig. 1). This phylogenetic context traverses the diversification of salmonid lineages and evolutionary origins of anadromy, a life-history strategy that is thought to have evolved at least twice independently [48] (Fig. 1) and potentially facilitated species diversification [7]. While the initially planned FAASG framework will hence enable high-resolution evolutionary reconstructions, additional taxa may be added as the salmonid research community progresses, potentially from the remaining genera (i.e. *Prosopium* within Coregoninae, *Parahucho* and *Brachymystax* within Salmoninae). FAASG will also address micro-evolutionary variation by contrasting wild populations that evolved divergent phenotypes over thousands of years and aquaculture vs. wild strains separated by a small number of generations (Fig. 1). The combination of experimental assays and evolutionary analyses done across the salmonid phylogeny (section Data and assays) will be applied to assess ‘genome function’ , thereby addressing a potential shortcoming of the original interpretations of the ENCODE data [49].

## Data and assays

The assays being considered for FAASG are described in Table 1 (also, see Additional file 1: Table S1). Annotating distinct classes of sequence variation will identify the genome-wide evolution of orthologous protein-coding genes, along with the large number of retained functional gene duplicates (>50% of those created) from WGD [9, 10]. Comparison of chromosome-anchored genome assemblies will provide insights into chromosomal re-arrangements accompanying rediploidization (e.g. [9]) and its potential impact on lineage-specific evolution. Population-level sequence variation will inform the role of functional elements in recent phenotypic divergence and adaptation (Table 1). The inclusion of northern pike (Fig. 1) will enable the ancestral (non-duplicated) state of sequence variation to be inferred, including the direction of divergence between duplicated genes. Comparisons to more distantly related fish with well-annotated genomes, including zebrafish [50], three-spined stickleback *Gasterosteus aculeatus* [51], spotted gar *Lepisosteus oculatus* [52], European seabass *Dicentrarchus labrax* [53], and Asian seabass *Lates calcarifer* [54], will allow salmonid-specific changes to be contextualized in the broader framework of teleost evolution, especially with respect to an earlier WGD event that occurred in the teleost ancestor ~320–350 Ma (e.g. [55]).

Transcriptome and proteome phenotypes will be characterized for a panel of tissues and developmental stages, sampled from both sexes under common-garden conditions using standardized sampling and analytical protocols (e.g. RNA extraction, quality control (i.e. integrity and purity), library preparation, choice of sequencing platform, and bioinformatic analyses) that distinguish divergence in expression of duplicated loci [9, 10]. Discerning the regulation and evolution of transcript complexity (e.g. non-coding, miRNome and splice variants) will necessitate stranded approaches [56] and may be facilitated by capture of full-length transcripts through single molecule real-time sequencing [57]. Standardized proteome expression profiling will also be performed after experimental separation of different cellular fractions.

FAASG will implement genome-wide experimental assays being used or considered under FAANG [11] (Table 1, Additional file 1: Table S1), potentially including: 1) methylation at nucleotide-level resolution (several approaches available, e.g. [58, 59]), 2) chromosome accessibility and architecture (via ATAC-Seq [60], DNase I footprinting [61], or ChIP-seq approaches), 3) histone modifications (using ChIP-seq approaches [62, 63]), 4) genome conformation (via Hi-C [64]) and 5) transcription factor binding occupancy (via ChIP-seq approaches [65]). It is noted that the lack of salmonid-specific reagents and antibodies present an initial barrier to implementation of these protocols. Indeed some have yet to be employed in salmonids and thus significant effort in methodological development will be required (Additional file 1: Table S1). However, several studies have laid the groundwork for such efforts, and no technical limitations are expected given that these approaches rely on generic techniques and conserved features of molecular biology. Initial experiments in Atlantic salmon and rainbow trout will be conducted in the context of regulation across tissues and developmental stages. Assays incorporating different lineages, populations, and physiological manipulations will follow within the wider proposed comparative-phylogenetic framework. Targeted genome editing can subsequently be used to infer causality of sequence variants and functional genomic elements.

When planning experiments, the FAASG consortium will implement a number of measures to reduce the need for experimentation with animals. These include giving due consideration to alternatives to in vivo experimentation such as cell culture, use of power analyses to determine appropriate sample sizes, exploiting already published RNAseq and microarray datasets relevant to traits of commercial or evolutionary interest, and the running of various FAASG assays across the same individuals within a study, as much as practicable. The latter will also increase power for linking variation across different levels of genome functional annotation.

## Importance of standardized phenotypic data

Informative genome functional annotation will necessitate standardized measurement and recording of both ecologically and production-relevant traits (section Traits of crosscutting relevance: from aquaculture to evolution (and beyond)) and for the effects of plasticity [66] to be controlled. Comparisons of the genetic architectures for complex phenotypes are confounded not only by the environment in which traits are measured, but also by how those traits are quantified. We view common-garden experiments, performed under agreed standardized conditions and treatments, as central to the collection of high-quality phenotype data. Salmonids are well-suited for common-garden experiments as they possess external fertilization, high fecundity, and have high survival rates in captivity. In addition, facilities are widely available to raise large numbers of fish under a range of controlled experimental contexts. Such features also facilitate robust and powerful studies to dissect the quantitative genetic basis of complex traits, albeit seasonal spawning may present logistical challenges for experimental planning. The standardized recording of both ecologically and production-relevant phenotypes and cataloguing of functional and phenotypic responses, e.g. within the Gene Ontology framework are also high priorities. Standardised phenotypic assays will also help interpret the molecular basis of phenotypic variation observed in the numerous wild populations gained by long-term data series, e.g. [67].

## Operational structure, funding and research community engagement

The initial governance of FAASG is via a Secretariat that supports a steering group incorporating chairs of four working groups and facilitates interactions with key industry and funder representatives. The working groups are generally similar in nature to those in FAANG, and consist of (i) animals, samples and assays, (ii) metadata and data sharing, (iii) bioinformatics and data analysis, and (iv) phenotyping. Details of the FAASG governance structure and working groups can be found at https://www.faasg.org/faasg-working-groups/. As the FAASG initiative requires major engagement and buy-in from researchers, industry and national funding bodies to be able to deliver the ambitious, high-level goals outlined above, members will seek opportunities to link existing or future projects to FAASG, in addition to capitalising on funding calls specifically aimed at reference genome annotation. The initiative will promote inclusiveness among all stakeholders and draw in expertise in aquaculture, bioinformatics/biostatistics, genetics, molecular biology, functional genomics, physiology, ecology and conservation, ensuring quality at all levels. For example, the second FAASG workshop in January 2017 in San Diego had 55 participants from 10 countries, including representatives of several funding bodies. The FAASG website (​https://www.faasg.org/) will report progress, including experimental and computational protocols, publications and datasets, along with contact information for interested researchers or funders who are invited to register on the same site. In addition, the initiative is being advertised at several scientific conferences to promote wider awareness.

## Additional file

Additional file 1: Table S1. Review of FAANG core/additional assays and relevant work performed in salmonids to date. (DOCX 37 kb)

## Abbreviations

FAANG: Functional Annotation of Animal Genomes
FAASG: Functional Annotation of All Salmonid Genomes
WGD: Whole genome duplication
