# Supplementary material for: Functional Annotation of All Salmonid Genomes (FAASG): an international initiative supporting future salmonid research, conservation and aquaculture
Source: BMC Genomics. 2017 Jun 27;18:484. doi: 10.1186/s12864-017-3862-8 (PMC5488370; doi:10.1186/s12864-017-3862-8)
Supplement: Additional file 1: Table S1. — Review of FAANG core/additional assays and relevant work performed in salmonids to date. (DOCX 37 kb) [file 12864_2017_3862_MOESM1_ESM.docx]

Table S1. **Review of FAANG core/additional assays and relevant work performed in salmonids to date**

| **FAANG core assays** | **Assay target** | **Example related assays used in salmonids; assay target** |
| --- | --- | --- |
| **Transcribed loci** | | |
| RNA-sequencing, stranded protocols [56, 70] | Transcriptome, strand polarity retained | RNA-seq, stranded protocol; transcriptome [71]  RNA-seq; double stranded; transcriptome [67-69]  RNA-seq; double stranded, miRNA [72]  RNA-seq; double stranded; Long non-coding RNA [73]  RNA-seq; double stranded; Large intergenic non-coding RNAs [74] |
| **Chromatin accessibility and architecture** | | |
| Assay for transposase-accessible chromatin sequencing (ATAC-seq) [[60, 75] | Regions of open chromatin, localization of nucleosomes in regulatory sites and positions of DNA-binding proteins | No published examples |
| DNaseI footprinting [59] | Open chromatin, delineate genomic regulatory compartments | No published examples |
| Chromatin immunoprecipitation sequencing (ChIP-seq) | Proteins linking genome architecture to function (FAANG- highly conserved insulator-binding factor, CTCF) [74] | No published examples |
| **Histone modification marks** | | |
| Chromatin immunoprecipitation sequencing (ChIP-seq) to detect modified histones and characterize associated sequences [61] | Histone H3 lysine 4 trimethylation (H3K4me3), identifies active gene promotors and is enriched at transcription start sites  Histone H3 lysine 27 trimethylation (H3K27me3), marks genes that have been facultatively repressed through regional modification  Histone H3 lysine 27 acetylation (H3K27ac), marks active regulatory elements, may discriminate active from inactive enhancers and promoters  H3 lysine 4 monomethylation (H3K4me1), marks enhancers and other distal elements, and is enriched downstream of transcription start sites | Chromosome immune precipitation; relationship between modified histones and gene expression [77] |
| **Additional FAANG Assays** | **Assay target** | **Related assay used in salmonids; assay target** |
| DNA methylation, genome-wide analysis of 5-methylcytosines, nucleotide level resolution [58-59] | Epigenetic mark and regulator of gene expression | Methyl-sensitive AFLP, global methylation changes [78-80]  Bisulphite sequencing, nucleotide-level resolution [82] |
| ChIP-seq assays [65] for sequences bound by specific proteins | Transcription factor binding sites | Chromosome Immune Precipitation Assay, regulation of transcription [83-85] |
| Genome conformation; Hi-C [64, 86] for chromosomal conformation capture | Identify distal chromatin elements that are brought together through 3D chromosomal folding | No published examples |

**Additional supporting references**

67. Koop BF, von Schalburg KR, Leong J, Walker N, Lieph R, Cooper GA et al. A salmonid EST genomic study: genes, duplications, phylogeny and microarrays. BMC Genomics. 2008, 9(1):1-16.

68. Pasquier J, Cabau C, Nguyen T, Jouanno E, Severac D, Braasch I et al. Gene evolution and gene expression after whole genome duplication in fish: the PhyloFish database. BMC Genomics 2016, 17(1):1-10.

69. Salem M, Paneru B, Al-Tobasei R, Abdouni F, Thorgaard GH, Rexroad CE et al. Transcriptome assembly, gene annotation and tissue gene expression atlas of the rainbow trout. PLoS ONE. 2015, 10(3):e0121778.

70. Mills JD, Kawahara Y, Janitz M. Strand-specific RNA-seq provides greater resolution of transcriptome profiling. Curr Genomics. 2013, 14(3):173-81.

71. Narum SR, Campbell NR. Transcriptomic response to heat stress among ecologically divergent populations of redband trout. BMC Genomics 2015, 16(1):1-12.

72. Andreassen R, Worren MM, Høyheim B. Discovery and characterization of miRNA genes in Atlantic salmon (*Salmo salar*) by use of a deep sequencing approach. BMC Genomics. 2013, 14:482-82.

73. Al-Tobasei R, Paneru B, Salem M. Genome-wide discovery of long non-coding RNAs in rainbow trout. PLoS One. 2016;11(2):e0148940.

74. Wang J, Fu LY, Koganti PP, Wang L, Hand JM, Ma H, Yao JB. Identification and functional prediction of large intergenic noncoding RNAs (lincRNAs) in rainbow trout (*Oncorhynchus mykiss*). Mar Biotechnol. 2016, 18(2):271-282.

75. Buenrostro J, Wu B, Chang H, Greenleaf W. ATAC-seq. A method for assaying chromatin accessibility genome-wide. Curr Protoc Mol Biol. 2015;109:21.29.1-9.

76. Ong CT, Corces VG. CTCF: an architectural protein bridging genome topology and function. Nat Rev Genet. 2014, 15(4):234-46.

77. Seiliez I, Froehlich JM, Marandel L, Gabillard JC, Biga PR: Evolutionary history and epigenetic regulation of the three paralogous pax7 genes in rainbow trout. Cell Tissue Res. 2015, 359(3):715-27.

78. Blouin MS, Thuillier V, Cooper B, Amarasinghe V, Cluzel L, Araki H et al. No evidence for large differences in genomic methylation between wild and hatchery steelhead (*Oncorhynchus mykiss*). Can J Fish Aquat Sci. 2010, 67(2):217-24.

79. Moran P, Perez-Figueroa A. Methylation changes associated with early maturation stages in the Atlantic salmon. BMC Genet. 2011, 12.

80. Moran P, Marco-Rius F, Megias M, Covelo-Soto L, Perez-Figueroa A. Environmental induced methylation changes associated with seawater adaptation in brown trout. Aquaculture 2013, 392:77-83.

81. Covelo-Soto L, Leunda PM, Perez-Figueroa A, Moran P: Genome-wide methylation study of diploid and triploid brown trout (*Salmo trutta* L.). Anim Genet. 2015, 46(3):280-88.

82. Baerwald MR, Meek MH, Stephens MR, Nagarajan RP, Goodbla AM, Tomalty KMH et al. Migration-related phenotypic divergence is associated with epigenetic modifications in rainbow trout. Mol Ecol. 2016, 25(8):1785-00.

83. Dann SG, Allison WT, Veldhoen K, Johnson T, Hawryshyn CW. Chromatin immunoprecipitation assay on the rainbow trout opsin proximal promoters illustrates binding of NF-kappa B and c-jun to the SWS1 promoter in the retina. Exp Eye Res. 2004, 78(5):1015-24.

84. Vuori KA, Nordlund E, Kallio J, Salakoski T, Nikinmaa M. Tissue-specific expression of aryl hydrocarbon receptor and putative developmental regulatory modules in Baltic salmon yolk-sac fry. Aquat Toxicol 2008, 87(1):19-27.

85. Lo JH, Chen TT. CCAAT/enhancer binding protein beta2 is involved in growth hormone-regulated insulin-like growth factor-II gene expression in the liver of rainbow trout *(Oncorhynchus mykiss*). Endocrinology 2010, 151(5):2128-39.

86. van Berkum NL, Lieberman-Aiden E, Williams L, Imakaev M, Gnirke A, Mirny LA et al.: Hi-C: A method to study the three-dimensional architecture of genomes. J Vis Exp. 2010;(39).
